# Implementing a structured model for osteoarthritis care in primary healthcare: A stepped-wedge cluster-randomised trial

**DOI:** 10.1371/journal.pmed.1002949

**Published:** 2019-10-15

**Authors:** Nina Østerås, Tuva Moseng, Leti van Bodegom-Vos, Krysia Dziedzic, Ibrahim Mdala, Bård Natvig, Jan Harald Røtterud, Unni-Berit Schjervheim, Thea Vliet Vlieland, Øyvor Andreassen, Jorun Nystuen Hansen, Kåre Birger Hagen

**Affiliations:** 1 National Advisory Unit on Rehabilitation in Rheumatology, Department of Rheumatology, Diakonhjemmet Hospital, Oslo, Norway; 2 Department of Biomedical Data Sciences, Leiden University Medical Center, Leiden, The Netherlands; 3 Primary Care Centre Versus Arthritis, School of Primary, Community and Social Care, Keele University, Keele, United Kingdom; 4 Department of General Practice, Institute of Health and Society, University of Oslo, Oslo, Norway; 5 Department of Orthopaedic Surgery, Akershus University Hospital, Lørenskog, Norway; 6 Health and Social Services, Nes Municipality, Norway; 7 Department of Orthopaedics, Leiden University Medical Center, Leiden, The Netherlands; 8 Patient Research Panel, Department of Rheumatology, Diakonhjemmet Hospital, Oslo, Norway; Univ. Paris Descartes, PRES Sorbonne Paris Cité, Hôpital Cochin, Assistance Publique - Hôpitaux de Paris, FRANCE

## Abstract

**Background:**

To improve quality of care for patients with hip and knee osteoarthritis (OA), a structured model for integrated OA care was developed based on international recommendations. The objective of this study was to assess the effectiveness of this model in primary care.

**Methods and findings:**

We conducted a cluster-randomised controlled trial with stepped-wedge cohort design in 6 Norwegian municipalities (clusters) between January 2015 and October 2017. The randomised order was concealed to the clusters until the time of crossover from the control to the intervention phase. The intervention was implementation of the SAMBA model, facilitated by interactive workshops for general practitioners and physiotherapists with an update on OA treatment recommendations. Patients in the intervention group attended a physiotherapist-led OA education and individually tailored exercise programme for 8–12 weeks. The primary outcome was patient-reported quality of care (OsteoArthritis Quality Indicator questionnaire; 0–100, 100 = optimal quality) at 6 months. Secondary outcomes included patient-reported referrals to physiotherapy, magnetic resonance imaging (MRI), and orthopaedic surgeon consultation; patients’ satisfaction with care; physical activity level; and proportion of patients who were overweight or obese (body mass index ≥ 25 kg/m^2^). In all, 40 of 80 general practitioners (mean age [SD] 50 [12] years, 42% females) and 37 of 64 physiotherapists (mean age [SD] 42 [8] years, 65% females) participated. They identified 531 patients, of which 393 patients (mean age [SD] 64 [10] years, 71% females) with symptomatic hip or knee OA were included. Among these, 109 patients were recruited during the control periods (control group), and 284 patients were recruited during interventions periods (intervention group). The patients in the intervention group reported significantly higher quality of care (score of 60 versus 41, mean difference 18.9; 95% CI 12.7, 25.1; *p <* 0.001) and higher satisfaction with OA care (odds ratio [OR] 12.1; 95% CI 6.44, 22.72; *p <* 0.001) compared to patients in the control group. The increase in quality of care was close to, but below, the pre-specified minimal important change. In the intervention group, a higher proportion was referred to physiotherapy (OR 2.5; 95% CI 1.08, 5.73; *p =* 0.03), a higher proportion fulfilled physical activity recommendations (OR 9.3; 95% CI 2.87, 30.37; *p <* 0.001), and a lower proportion was referred to an orthopaedic surgeon (OR 0.3; 95% CI 0.08, 0.80; *p =* 0.02), as compared to the control group. There were no significant group differences regarding referral to MRI (OR 0.6; 95% CI 0.13, 2.38; *p =* 0.42) and proportion of patients who were overweight or obese (OR 1.3; 95% CI 0.70, 2.51; *p =* 0.34). Study limitations include the imbalance in patient group size, which may have been due to an increased attention to OA patients among the health professionals during the intervention phase, and a potential recruitment bias as the patient participants were identified by their health professionals.

**Conclusions:**

In this study, a structured model in primary care resulted in higher quality of OA care as compared to usual care. Future studies should explore ways to implement the structured model for integrated OA care on a larger scale.

**Trial registration:**

ClinicalTrials.gov NCT02333656.

## Author summary

**Why was this study done?:**

- Hip and knee osteoarthritis is a common chronic joint disease in the adult population causing significant pain and disability.
- Non-surgical treatment modalities including patient osteoarthritis education, exercise therapy, and weight management represent core treatments recommended in professional guidelines. However, they are currently underutilised in people with hip and knee osteoarthritis.
- It is not established to what extent a structured osteoarthritis care model can change this and improve the quality of care.

**What did the researchers do and find?:**

- A cluster-randomised trial was conducted to compare a structured osteoarthritis care model with usual care with respect to appropriate care delivery in people with hip and knee osteoarthritis.
- Forty general practitioners and 37 physiotherapists working in primary care attended workshops to get an update on recommendations for osteoarthritis care and were trained in the core elements of the structured care model: osteoarthritis education in groups, an individually tailored 8- to 12-week exercise programme, and a dietary intervention, if needed.
- Of the 393 patient participants, 284 were allocated to the intervention group and 109 to the usual care group.
- At 6 months, patient-reported quality of care and satisfaction with care were greater, more patients were referred to physiotherapy and fewer to orthopaedic surgeons, and more patients fulfilled physical activity criteria in the intervention group as compared to the usual care group.

**What do these findings mean?:**

- A structured osteoarthritis care model provided by trained primary care general practitioners and physiotherapists resulted in the provision of osteoarthritis care that was more in line with current care recommendations and in higher patient-reported quality of care and satisfaction as compared to usual care.
- A structured and well-planned approach, in line with evidence-based treatment recommendations for hip and knee osteoarthritis and executed in primary care, has the potential to improve patients’ health and reduce disability. In doing so, it may also reduce the risk of sick leave and may thereby reduce the direct and indirect costs of osteoarthritis for the individual and the society.
- Although a stepped-wedge cluster-randomised controlled trial design is appropriate to conduct an effectiveness study in a clinical practice setting, strategies to prevent selection bias and differences in recruitment rates in the control and intervention periods are needed.

## Introduction

Osteoarthritis (OA) is one of the leading causes of pain and disability in the adult population worldwide and a major contributor to years lived with disability [1,2]. Prevalence of OA increases with age, and with an aging population and the epidemic of obesity, it is set to rise [3]. The costs of treatment and work-related losses represent a considerable economic burden [4,5]. Recommended first-line core treatments include patient education, self-management, exercise, and weight reduction [1,6–8]. When non-pharmacological and pharmacological care fail, joint replacement offers an effective approach, although it is costly and associated with medical and surgical risks [9–12]. The demand for joint replacement is expected to accelerate and quadruple by 2030 with the increasing prevalence of OA [13]. Decisions on joint replacement involve conventional radiographs, whereas magnetic resonance imaging (MRI) is usually considered unnecessary [14].

An evidence-to-practice gap for OA care has been identified internationally, with poor uptake of non-pharmacological approaches such as patient education and exercise treatment in contrast to surgical treatment [15,16]. Furthermore, as 22%–68% of joint replacements are considered inappropriate [17], it is important to improve the uptake of non-surgical care. A small number of best practice initiatives to improve the quality of OA care have shown promising but somewhat diverging results [18–24]. Inspired by these previous initiatives [18–25] and based on international recommendations for OA care [1,6–8], the SAMBA model for integrated care for patients with hip and knee OA [26] was developed by the research team for evaluation in a randomised controlled trial. SAMBA is an acronym formed from the Norwegian project title, ‘SAMhandling for Bedre Artrosebehandling i kommunehelsetjenesten’, which can be translated as ‘improved management of patients with hip and knee osteoarthritis in primary healthcare’.

The main aim of the present study was to assess the effectiveness of the SAMBA model in primary healthcare. We hypothesised that compared to usual OA care, the SAMBA model would increase the uptake of best practices for OA, demonstrate higher patient satisfaction with care, and increase beneficial lifestyle characteristics (physical activity, healthy weight). We hypothesised that compared with usual care, the SAMBA model would offer improvements in referral pathways, e.g., more general practitioner (GP) referrals to physiotherapy, more discharge reports from physiotherapy to referring GPs, and fewer GP referrals to MRI and orthopaedic surgeons.

## Methods

### Design, setting, and participants

**Fig 1:**
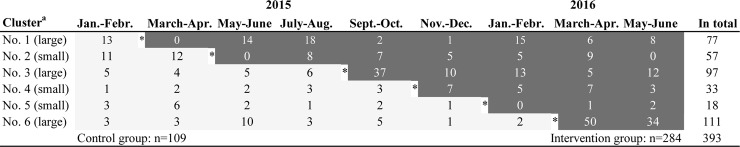
Stepped-wedge design, timeline, and patient recruitment rate. All 6 municipalities (clusters) started the trial simultaneously with a control phase (general practitioners and physiotherapists providing usual care). At predefined time points about every second month, one by one the municipalities crossed from the control to the intervention phase (use of the SAMBA model) in a randomised order. Light cells in the figure represent control periods, and dark cells represent intervention periods. The asterisks indicate the timing of the interactive workshops before switching to the intervention phase. Patients recruited to the study during the control phase in any cluster constituted the control group, whereas patients recruited during the intervention phase constituted the intervention group. All patients responded to the baseline questionnaire and follow-up questionnaires at 3, 6, 9, and 12 months post-baseline. ^a^Large municipalities (clusters) had >20,000 inhabitants.

We performed a cluster-randomised controlled trial (cluster-RCT) with a stepped-wedge cohort design between 15 January 2015 and 20 October 2017. The study was conducted in 6 neighbouring municipalities (clusters) north of Oslo, Norway, with approximately 100,000 inhabitants in total. The stepped-wedge design is explained in Fig 1 and in the published study protocol [26]. The study was prospectively registered at ClinicalTrials.gov (NCT02333656) and is reported according to the CONSORT (S1 Text) and TIDieR (S2 Text) checklists [27,28].

The Regional Committee for Medical and Health Research Ethics issued a letter of exemption for the current study (Ref. No: 2014/1739 REK south-east C). The Data Inspectorate/Data Protection Official of Oslo University Hospital approved the study on 22 December 2014. Written informed consent was obtained from patients upon inclusion.

All GPs and physiotherapists (PTs) working in private practice or healthy life centres in the 6 municipalities were invited to participate. Healthy life centres provide primary-care-based services aiming to support a healthy lifestyle for people with chronic diseases [29].

Potential eligible patients were identified at clinical visits by the GPs and PTs using the following inclusion criteria: age ≥ 45 years with symptomatic hip and/or knee OA diagnosis verified clinically or radiologically by the GP. Patients who did not understand Norwegian or who had 4 joint replacements (hip + knee), inflammatory rheumatic disease, malignant illness, or any other major condition that restricted their ability to adhere to the intervention were excluded [26]. A study coordinator performed the eligibility screening and inclusion procedure.

### Randomisation and blinding

Immediately before study initiation, the municipalities were randomly allocated to 1 of the 6 sequences for time of crossover from the control to the intervention phase (Fig 1) using a computer-generated list of random numbers provided by a statistician. To ensure a mix of municipality sizes in the randomised sequence, stratification on the number of inhabitants (less than versus more than 20,000) was performed. The randomised order was concealed to the clusters until soon before the switch. It was not possible to blind the involved GPs, PTs, or patients, but a statistician blinded for group allocation performed the statistical analyses of the primary outcome.

### Intervention

**Fig 2:**
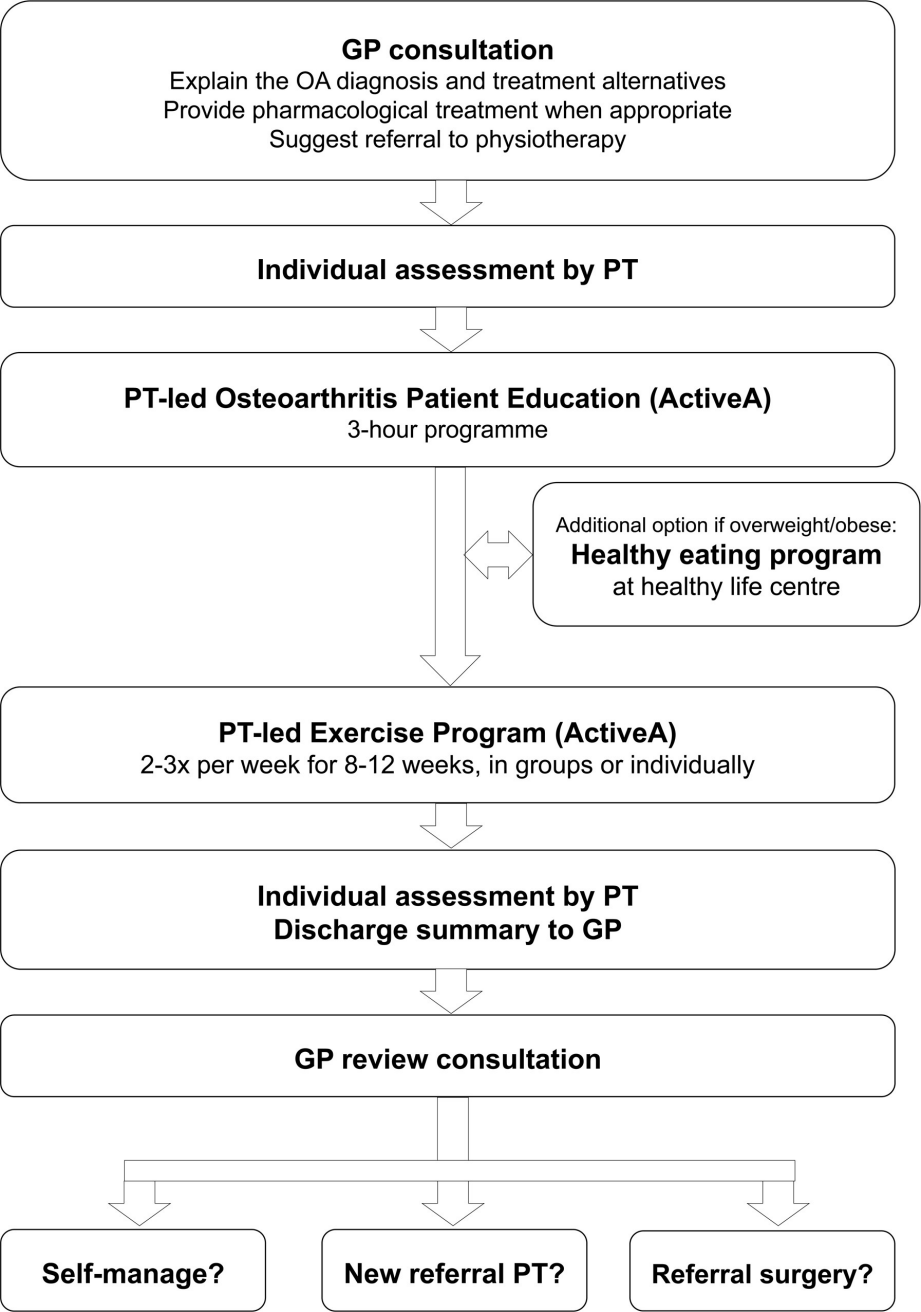
The SAMBA model for integrated osteoarthritis care. GP, general practitioner; OA, osteoarthritis; PT, physiotherapist.

The SAMBA model for integrated OA care was developed by the research team and comprised a structured pathway for patients with OA through the healthcare system (Fig 2). The model included a GP consultation, a PT-led OA education and exercise programme (ActiveA), an optional healthy eating program, and a GP review consultation. The GPs were instructed to explain the OA diagnosis and treatment alternatives, provide pharmacological treatment when appropriate, and suggest referral to physiotherapy. The PT-led patient OA education programme was group-based and lasted 3 hours. This was followed by an 8–12 week exercise programme with twice weekly 1-hour supervised group sessions (5–10 patients per PT). Based on patient examination, the PT prescribed individually tailored resistance exercise programmes to increase muscular strength. The pool of recommended exercises was selected from previous OA exercise studies [21,22,30,31]. Dose recommendations were based on acknowledged international guidelines [32], and included gradually increasing the dose towards 2–4 sets with 8–12 repetitions and 60%–70% of 1 repetition maximum, or more if tolerated. The PTs were instructed to closely monitor the patients’ exercise performance and regularly provide appropriate individual adjustments of the exercise programme for progression. When the patient could perform 2 extra repetitions in the last set, the resistance was increased (‘the 2+ principle’). The patients were encouraged to add a third home-based session consisting of 30–60 minutes of cardiorespiratory exercise like brisk walking, running, or bicycling.

The model intends to improve the quality of OA care through delivery of evidence-based recommendations for OA care, focusing on the core treatments, first-line analgesics, and facilitating multidisciplinary collaboration. Informed by theoretical models [33,34] and previously developed behaviour change interventions [35], a strategy [26] was designed to facilitate the use of the model among GPs and PTs in primary healthcare in the intervention phase (S3 Text). Tailored interactive workshops for GPs and PTs were arranged in close proximity to the time of crossover (Fig 1) and reflected the main intervention, ensuring the use of the model in the intervention phase. Other strategies to facilitate the use of the SAMBA model in the intervention phase included summarised treatment recommendations, regular telephone reminders, quarterly letters with feedback, and biannual outreach visits known to be effective in primary care (see details in the published protocol [26]).

#### The PT workshop

The PT workshop included a 1-day (9 hours) workshop-based education programme organised by the Active with osteoArthritis (ActiveA) programme [36], which builds on the similar Swedish [21] and Danish [22] programmes. The workshop included an update on OA epidemiology, clinical features, and treatment recommendations. Education in delivery of a patient OA education programme, individually tailored semi-standardised exercises, performance testing, and healthy eating and weight reduction strategies was given. The PTs received access to the ready-to-use patient OA education programme (PowerPoint file and manuscript) and access to a database with recommendations for resistance exercises and dose.

#### The multidisciplinary workshop

The 1.5-hour multidisciplinary (GPs and PTs) workshops were conducted within the general practices at established meeting time points in order to facilitate high GP attendance. The workshops included an update on current treatment recommendations. An orthopaedic surgeon presented views on when to consider referral to consultation with an orthopaedic surgeon, emphasising the importance of exploiting conservative treatment first. The research team presented the SAMBA model and facilitated a multidisciplinary discussion regarding OA care.

Attendance at workshops, patient adherence to the OA education and exercise programme, and potential adverse effects were captured from study records and patient-reported exercise diaries. A comprehensive analysis of fidelity has been published in a separate article [37], and the other secondary outcomes will be reported separately.

### Control

During the control phase, the GPs and the PTs delivered usual care and were naïve to the SAMBA model. Usual care may include infrequent GP visits, pharmacological therapy, and occasionally a referral to physiotherapy. Patients included during the control phase were allowed to receive physiotherapy, but not to attend the patient OA education programme nor the individually tailored exercise programme, prior to 12 months post-baseline. Physiotherapy provided to patients with knee OA may include exercise, but also often includes several other treatment modalities (e.g., massage, traction/mobilisation, stretching, and electrotherapy) showing moderate or low quality of evidence, or no evidence, in systematic reviews [38].

### Data collection

Patients self-reported at baseline (shortly after the GP consultation) and at 3 months (T_3_) and 6 months (T_6_) using an electronic questionnaire or mailed paper questionnaire returned in a prepaid envelope. The primary time point was T_6_, except for referral to physiotherapy (T_3_). Data on long-term follow-up (9 and 12 months) will be published later. Information on the patients’ age, sex, previous or planned joint replacements, and comorbidity was collected by the study coordinator during telephone screening. Other patient characteristics and OA-disease-related information, including the Knee injury and Osteoarthritis Outcome Score/Hip disability and Osteoarthritis Outcome Score Activities of Daily Living subscale (KOOS/HOOS ADL subscale), were self-reported at baseline. The GPs and PTs self-reported demographics and practice information in a questionnaire.

### Primary outcome measure

The primary outcome was patient-reported quality of OA care at T_6_ measured with the OsteoArthritis Quality Indicator questionnaire version 2 (OA-QI v2) (S4 Text) [39]. OA-QI v1 was developed in 2010 based on published quality indicators (QIs) for OA care identified in a literature search and further refined via expert panels and patient interviews [40], and was slightly revised in 2015 [39]. OA-QI v2 reflects current OA care guideline recommendations [1,6–8] and includes 16 QI items related to patient OA education and information, regular provider assessments, referrals, and pharmacological treatment. An example of an item with response alternatives is as follows: ‘Have you been given information about osteoarthritis from a health professional? Yes/No/Don’t remember’.

All items have ‘Yes’, ‘No’, and ‘Not applicable’/‘Don’t remember’ as response options. Each QI item was considered passed if the patient had checked ‘Yes’ and was considered ‘eligible’ if the patient responded ‘Yes’ or ‘No’ for that item. On the patient level, the QI pass rate was calculated as the total number of items passed divided by the number of eligible items for each patient (in percentage), ranging from 0 to 100, with 100 representing the best quality of care score. On the group level, the mean total pass rate was calculated.

Previous applications of the questionnaire have showed acceptable measurement properties including reliability, validity, responsiveness, and interpretability [39,40]. The test–retest reliability for the total pass rate was acceptable (intraclass correlation coefficient [ICC] 0.89) [39]. All predefined hypotheses to assess construct validity were confirmed, and responsiveness was acceptable, with 3 of 4 predefined hypotheses confirmed [39]. Minimal important change for the total pass rate was assessed to be 20.4 on the 0 to 100 scale [39]. OA-QI v1 has been previously tested in UK primary care in a cluster-randomised trial and has been shown to be responsive to the use of national recommendations for OA care [23].

### Secondary outcome measures

GP referrals to PTs, MRI, and orthopaedic surgeons were patient self-reported as ‘Yes’ or ‘No’.

The Norwegian Health Economics Administration provided data on the total number of registered discharge reports for all patients for participating PTs during the control and intervention phases. This number may also include discharge reports related to non-participating patients and should be interpreted with caution.

Patients’ satisfaction with OA care was assessed using 1 item, with 5 response alternatives ranging from ‘Very satisfied’ to ‘Very dissatisfied’, from a previous study [41].

Physical activity was reported using 3 items on frequency (never/less than once per week/once per week/2 to 3 times per week/4 or more times per week), duration (less than 15 minutes/15–30 minutes/31–60 minutes/more than 60 minutes), and intensity (no sweat/sweat [moderate]/exhausted [vigorous]) [42]. Using an index from a previous study [43], we calculated the proportion of patients ‘fulfilling’ versus ‘below’ recommendations. Corresponding to the physical activity recommendations at the time of that study, ‘fulfilling’ was 150 minutes of moderate-intensity activity or 60 minutes of vigorous-intensity activity each week, or a combination of these.

The proportion of patients who were overweight (body mass index [BMI] ≥ 25 kg/m^2^) or obese (BMI ≥ 30 kg/m^2^) was defined using patients’ self-reported body height at baseline and body weight at follow-ups.

Other patient-level secondary outcomes (e.g., symptoms, function, quality of life, and performance tests) and an economic evaluation will be reported later.

### Sample size calculation

Based on previous research [44], we estimated the ICC to be <0.01. We estimated that a minimum of 194 individuals in each group among the 6 clusters, with an average of 50 individuals per cluster, would achieve 80% power to detect a 10-unit difference between the group means on the primary outcome measure, where standard deviation for the primary outcome measure was 24 units and intra-class correlation was 0.01, using a 2-sided test with a significance level of 0.05. To account for 30% patient dropout, we aimed to include 388 patients in total.

### Statistical analyses

The primary outcome analysis was performed on an intention to treat basis by comparing OA-QI v2 mean total pass rate in the control versus the intervention group. Multilevel mixed models with random intercepts were fitted to adjust for the effect of clustering (municipality), participant (patient), and repeated measures over time. This model accounts for dropout under a missing at random assumption. The primary outcome was assessed with a linear model, whereas the secondary outcomes were assessed with logistic models except for patient satisfaction with OA care, which was assessed applying multilevel ordered logistic regression models. All regression models included an interaction term of follow-up time point and group, and were adjusted for age, sex, and secular time (number of months between study initiation and the patient entering the study). Statistical analyses were performed with STATA/IC 14.

### Patient and public involvement and engagement

Patient research partners were involved in all stages of this trial, from grant application, development of study materials (including questionnaire and consent procedure) and intervention package, and interpretation of the results to final dissemination of results. Two patient research partners (ØA and JNH) were members of the trial steering committee and are co-authors of the present article.

## Results

**Fig 3:**
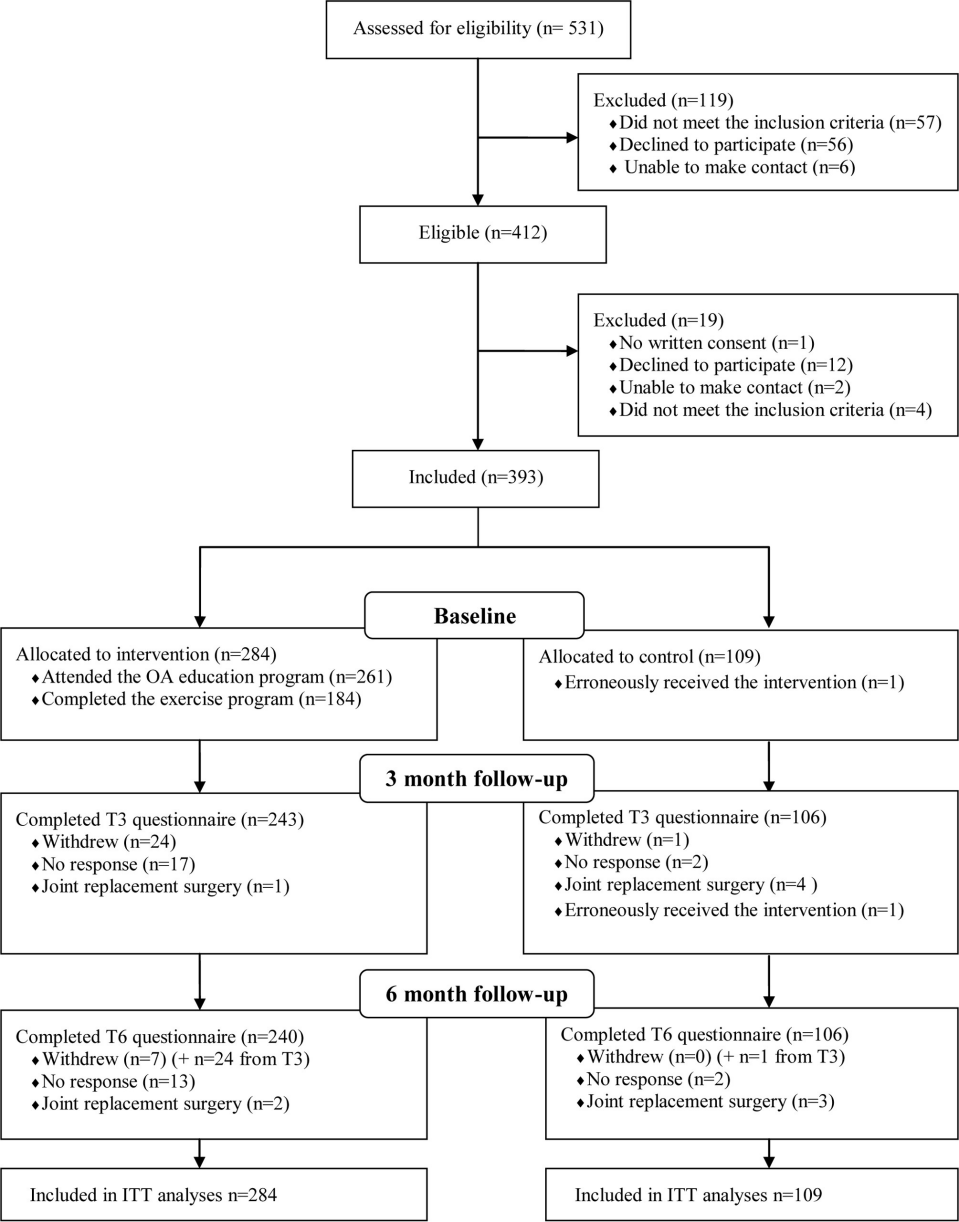
CONSORT patient flow diagram. ITT, intention to treat; OA, osteoarthritis.

Forty (50%) of the 80 GPs and 37 (50%) of the 64 PTs in the 6 municipalities (clusters) attended the workshops. Of the 531 patients identified by these GPs and PTs, 393 (74%) fulfilled the inclusion criteria and were willing to participate (Fig 3). Patients who were excluded (*n =* 138) were similar to patients who were included in terms of sex and age. In total, 109 patients (control group) were recruited during the control periods across the clusters, and 284 patients (intervention group) were recruited during interventions periods (Fig 1). Baseline characteristics of the patients, GPs, and PTs are provided in Table 1. In total, 89% of the patients (*n =* 349) completed the T_3_ questionnaire and 88% (*n =* 346) completed the T_6_ questionnaire (Fig 3). Patients who completed versus did not complete these questionnaires did not differ with regards to baseline characteristics.

**Table 1:** Baseline characteristics of patients, physiotherapists, and general practitioners.

Variable	Patients		Physiotherapists (*n =* 37)	General practitioners (*n =* 40)
	Intervention group (*n =* 284)	Control group (*n =* 109)		
**Sex, female, *n* (%)**	211 (74)	68 (62)	24 (65)	17 (42)
**Age, years, mean (SD)**	63 (10)	65 (10)	42 (8)	50 (12)
**BMI, kg/m**^**2**^**, mean (SD)**	29 (6)	28 (5)		
**Education at university level, *n* (%)**	101 (36)	35 (32)		
**Work status, *n* (%)**				
Full- or part-time employed	108 (38)	37 (34)		
Age retired	112 (39)	50 (46)		
On sick leave	15 (5)	5 (5)		
Receiving disability pension	31 (11)	11 (10)		
Other	20 (7)	6 (6)		
**Main affected joint, *n* (%)**				
Knee	174 (61)	54 (49)		
Hip	100 (35)	46 (42)		
Other	9 (3)	9 (8)		
**Hip or knee joint prosthesis, *n* (%)**				
No joint prosthesis	258 (91)	99 (91)		
1 joint	14 (5)	6 (6)		
2 joints	6 (2)	3 (3)		
3 joints	6 (2)	1 (1)		
**Years since OA diagnosis, mean (SD)**	7 (10)	7 (6)		
**Mean pain level past week, NRS 0–10, mean (SD)**	5.4 (2.0)	5.1(1.9)		
**Other chronic disease, yes, *n* (%)**	71 (25)	28 (26)		
**KOOS/HOOS ADL subscale, mean (SD)**	68 (20)	68 (20)		
**Work years, median (IQR)**			8 (2, 14)	8 (3, 23)
**Number of treated patients per day, mean (SD)**			12 (4)	21 (4)
**Exercise groups per week, mean (SD)**			2 (2)	
**Patient list size, mean (SD)**				1,130 (296)

BMI, body mass index; IQR, interquartile range; KOOS/HOOS ADL subscale, Knee injury and Osteoarthritis Outcome Score/Hip disability and Osteoarthritis Outcome Score Activities of Daily Living subscale (score range 0–100, 100 = best); NRS, numeric rating scale (0–10, 0 = no pain, 10 = worst pain); OA, osteoarthritis; SD, standard deviation.

In total, 27 PT-led patient OA education and exercise groups were arranged, with 92% (*n =* 261) of the patients in the intervention group attending the OA education programme, and 64% (*n =* 184) completing ≥8 weeks of the exercise programme. The completers versus non-completers among the intervention patients did not differ regarding sex or age.

Seven (6%) patients in the control and 3 (1%) in the intervention group received joint replacement surgery between baseline and T_6_. Four patients in the intervention group experienced increased prolonged knee pain and/or swelling and discontinued the exercise programme at the halfway stage. Two patients in the control group receiving physiotherapy (usual care) erroneously attended the PT-led education and exercise programme after their PT had attended the workshop.

### Primary outcome

**Fig 4:**
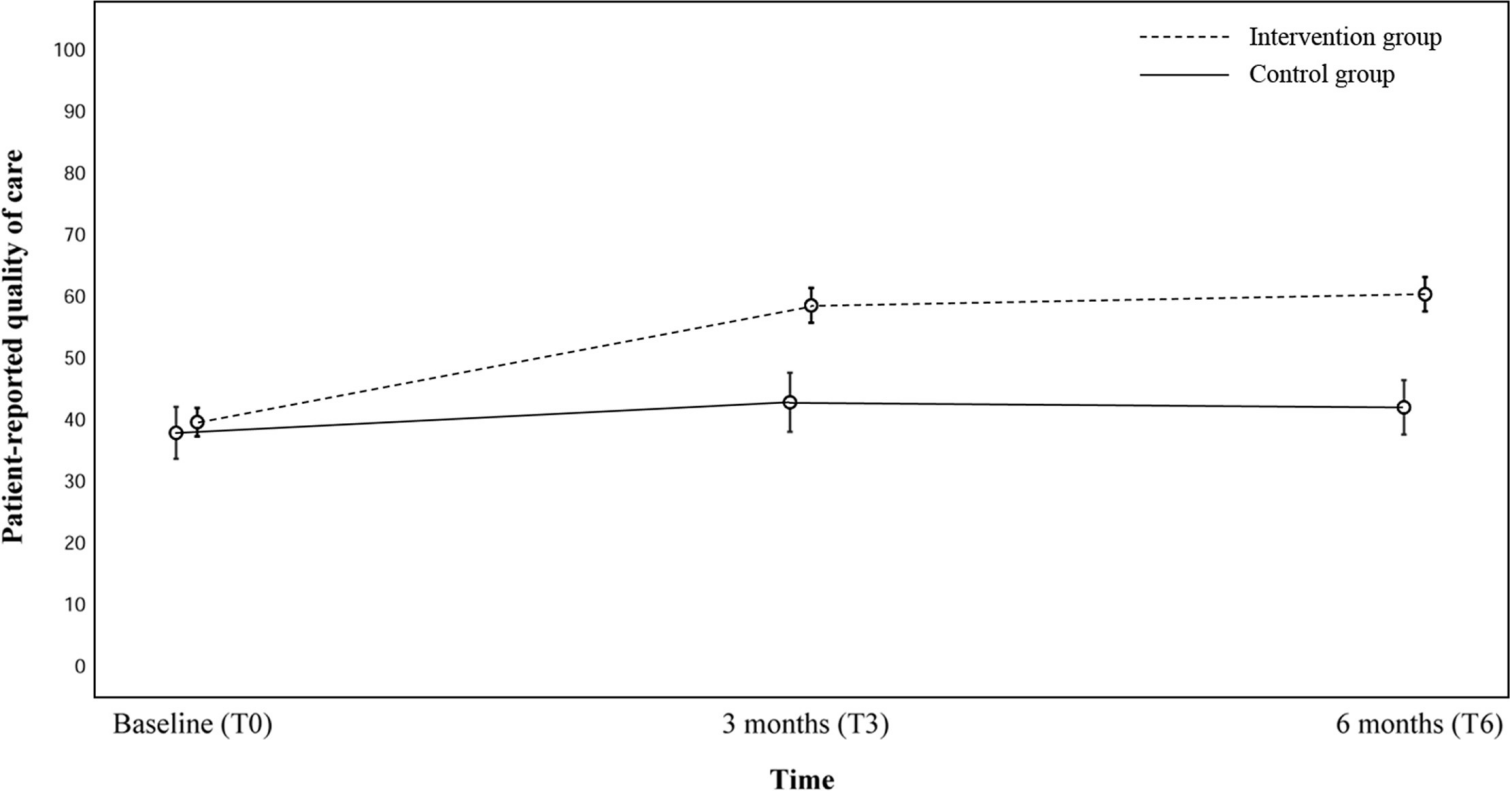
Mean patient-reported quality of care in the control group (*n =* 109) and intervention group (*n =* 284) at baseline and 3 and 6 months of follow-up. Mean patient-reported quality of care with 95% confidence interval. Patient-reported quality of care captured by OsteoArthritis Quality Indicator questionnaire version 2 (0–100, 100 = best score).

At baseline, the OA-QI v2 mean total pass rate was similar for the intervention (39%) and the control group (37%). At T_6_, the pass rate was higher (60%) for the intervention as compared to the control group (41%) (Table 2). This was due to a higher uptake of non-pharmacological treatment recommendations in the intervention group, and in particular the core treatments: patient education about the disease and treatment alternatives, self-management, and exercise (Table 2). Adjusted multilevel mixed model analyses showed a statistically significant difference in mean total pass rate at T_6_, with higher quality of care in the intervention as compared to the control group (mean difference 18.9; 95% CI 12.7, 25.1; *p <* 0.001) (Table 2; Fig 4). The crude ICC was 0.016.

**Table 2:** Primary outcome and individual item pass rates at baseline and 3 and 6 months of follow-up and mean difference between groups (*n =* 393).

Outcome measure	Control group			Intervention group			Control versus intervention group, mean difference (95% CI)	
	T_0_ *n =* 109	T_3_ *n =* 106	T_6_ *n =* 106	T_0_ *n =* 284	T_3_ *n =* 243	T_6_ *n =* 240	T_3_ *n =* 109 versus 284	T_6_ *n =* 109 versus 284
**Quality of OA care, OA-QI v2 mean total pass rate**^**a**^	37	42	41	39	58	60	16.5 (10.3, 22.6)**	18.9 (12.7, 25.1)**
**Individual OA-QI v2 item pass rates**^b^								
Information about OA from a HP	25	30	37	25	68	70		
Information about different treatment alternatives	46	35	42	34	69	74		
Information on self-management	24	35	36	26	75	80		
Information about importance of physical activity	69	76	74	62	95	98		
Referred to HP for physical activity/exercise	65	60	58	68	85	83		
Advised to lose weight	25	35	39	43	54	55		
Referred for support to lose weight	3	7	3	11	21	12		
Assessed for functional ability	17	15	22	19	29	29		
Assessed the need for walking aids	16	28	18	12	19	22		
Assessed the need for other aids	9	6	9	6	8	11		
Joint pain assessed by HP	53	53	48	59	63	64		
Paracetamol recommended as first line	54	63	67	62	65	66		
Offered stronger pain killers	40	43	39	42	47	44		
Information about anti-inflammatory medication	51	50	54	55	63	66		
Offered steroid injection	29	26	24	23	26	27		
Referred to orthopaedic surgeon	30	37	36	30	26	33		

**p < 0.001. Estimates are adjusted for patient age, sex, and secular time (number of months between study initiation and the patient entering the study).

^a^The mean total pass rate was calculated on the group level (from individual patients’ pass rates) as the mean percentage of QI items passed, ranging from 0 100, with 100 representing the best quality of care.

^b^Individual OA-QI v2 item pass rates were calculated as the proportion of patients reporting that the QI was passed divided by the proportion of patients who were eligible for that QI item (in percentage), ranging from 0 to 100, with 100 representing that all eligible patients reported pass for that QI item.

HP, health professional; OA, osteoarthritis; OA-QI v2, OsteoArthritis Quality Indicator questionnaire version 2; QI, quality indicator; T_0_, baseline; T_3_, 3-month follow-up; T_6_, 6-month follow-up.

### Secondary outcomes

At T_3_, a significantly higher proportion of patients in the intervention group reported PT referrals compared to the control group (36% versus 25%; odds ratio [OR] 2.5; 95% CI 1.08, 5.73; *p =* 0.03) (Table 3). The number of physiotherapy discharge reports was 59 for the control and 127 for the intervention periods. At T_6_, a negligibly lower proportion was referred to MRI (5% versus 8%; OR 0.6; 95% CI 0.13, 2.38; *p =* 0.42), but a significantly lower proportion was referred to an orthopaedic surgeon (6% versus 13%; OR 0.3; 95% CI 0.08, 0.80; *p =* 0.02), in the intervention as compared to the control group (Table 3).

**Table 3:** Secondary outcomes at baseline and 3 and 6 months of follow-up and odds ratio between groups (*n =* 393).

Outcome	Control group percent			Intervention group percent			Control versus intervention group, OR (95% CI)	
	T_0_ *n =* 109	T_3_ *n =* 106	T_6_ *n =* 106	T_0_ *n =* 284	T_3_ *n =* 243	T_6_ *n =* 240	T_3_ *n =* 109 versus 284	T_6_ *n =* 109 versus 284
**Referred to physiotherapy**	48	25	30	52	36	22	2.5 (1.08, 5.73)*	0.7 (0.28, 1.52)
**Referred to MRI**	27	14	8	26	10	5	0.6 (0.17, 2.34)	0.6 (0.13, 2.38)
**Referred to orthopaedic surgeon**	6	10	13	9	4	6	0.2 (0.06, 0.73)*	0.3 (0.08, 0.80)*
**Fulfilling physical activity recommendation**^**a**^	48	44	45	51	78	67	28.4 (8.30, 97.08)**	9.3 (2.87, 30.37)**
**Being overweight/obese**^**b**^	69	69	67	72	70	69	1.3 (0.69, 2.54)	1.3 (0.70, 2.51)

*p < 0.05

**p < 0.001. Estimates are adjusted for patient age, sex, and secular time (number of months between study initiation and the patient entering the study).

^a^To fulfil the physical activity recommendations, the patients had to report moderate-intensity activity for 150 minutes or vigorous-intensity activity for 60 minutes per week, or a combination of these.

^b^Overweight: BMI ≥ 25 kg/m^2^; obese: BMI ≥ 30 kg/m^2^.

BMI, body mass index; OR, odds ratio; T_0_, baseline; T_3_, 3-month follow-up; T_6_, 6-month follow-up.

Compared to the control group, the intervention group had a significantly higher OR for reporting satisfaction with OA care at T_6_ (OR 12.1; 95% CI 6.44, 22.72; *p <* 0.001). A significantly higher proportion in the intervention as compared to the control group (67% versus 45%) fulfilled the recommendation for weekly physical activity at T_6_ (OR 9.3; 95% CI 2.87, 30.37; *p <* 0.001) (Table 3). The proportion of patients who were overweight or obese remained similar in the 2 groups (69% versus 67%; OR 1.3; 95% CI 0.70, 2.51; *p =* 0.34) (Table 3).

## Discussion

The evidence-to-practice gap for OA care and the diverging results in previous studies aiming to improve OA care highlight the need for care models that increase adherence by practitioners to recommendations for OA care. This cluster-RCT assessing a structured model in primary healthcare is, to our knowledge, among the first to show an increased uptake of core OA treatment recommendations among GPs and PTs. Patients in the intervention group reported significantly higher quality of care than patients in the control group, and had better outcomes for 4 of the 6 secondary outcomes related to satisfaction with care, referral pathways, and beneficial lifestyles.

### Primary outcome

The observed between-group difference in OA-QI v2 mean total pass rate at 6-month follow-up (T_6_) indicates that the use of the structured OA care model successfully improved the delivery of OA care among GPs and PTs in primary healthcare in this study. While the mean total pass rate at baseline in this study was comparable to that in previous studies [15,16], the mean total pass rate at T_6_ for the intervention group was higher than the pass rates in most previous studies. The increase in mean total pass rate in the intervention group was comparable to the increase observed in a Norwegian longitudinal patient cohort study including a patient OA education programme [39]. Despite the significant between-group difference, the increase in mean total pass rate in the current study did not meet the minimal important change for this outcome measure, but was very close.

Among previous studies aiming to improve OA care, a UK cluster-RCT [23] implementing a model OA consultation in general practice and a Dutch randomised controlled trial [45] with interactive clinical workshops for PTs resulted in improved guideline adherence. The UK cluster-RCT [23] resulted in increased referral rates to physiotherapy, as did the current study. However, in a Dutch cohort study implementing a stepped OA care strategy in general practice, the provided care became only modestly consistent with the strategy [20]. This conflicting result may be due to differences in design, settings, and models to improve OA care. The current study and the UK cluster-RCT [23] had a multidisciplinary approach, whereas the 2 Dutch studies focused on either PTs [45] or GPs [20]. Targeting more than one health profession may add beneficial effects including improved multidisciplinary collaboration, integrated care, and consistent patient information.

The mean total pass rate in the intervention group at T_6_ indicates that there is a potential for even further improvements in provided care. Looking at individual quality of care items in OA-QI v2, the improvement was particularly evident for core treatment elements related to patient information, self-management, and exercise. This would be expected as these core elements were the main focus of the study intervention, and an improvement on all individual OA-QI v2 items would not be realistic in this study. The improvement for provision of core treatments is in line with the UK cluster-RCT, in which the intervention also resulted in higher uptake of core guideline recommendations [23]. By scrutinising the individual QI item pass rates, ideas for further improvements of the care model may be generated, e.g., regarding advice and support on weight reduction.

### Secondary outcomes

The study intervention had a positive effect on patient satisfaction with care, patient physical activity, and referral rates to PTs and orthopaedic surgeons. The complex intervention in this study means that there are many factors that could have contributed to the higher patient satisfaction with care, e.g., the structured approach to OA care or the updated knowledge among GPs and PTs on current OA care recommendations. Unfortunately, it is not possible to disentangle the effect of the different factors in this study. The increase in PT referrals and the decrease in referrals to orthopaedic surgeons demonstrate that the GPs in this study adhered to the structured OA care model and that the patient participants exploited the core treatments before considering referral for surgical consultation.

However, the intervention showed negligible effects on MRI referrals and on the proportion of patients who were overweight or obese. Regarding MRI, fewer referrals at T_6_ as compared to baseline were observed in the intervention group, but there was also a decrease in the control group. The reason for the decrease in the control group is unknown, but may be related to the relatively high referral rate at baseline for both groups, which reduced the number of candidates for MRI referrals at follow-up time points. Although the structured care model included an optional healthy eating program, an intensive diet may have been required to observe an effect on the patients’ BMI and the proportion of patients who were overweight or obese over the time period studied here [46].

### Strengths and limitations

The study has several strengths including the robust but pragmatic study design, the multidisciplinary approach, and the large patient sample. The stepped-wedge design was chosen since it allowed all clusters to test the intervention, and the GP and PT training could be done in one cluster at the time over a longer period. However, the complexity of the stepped-wedge design makes reporting of study methods and results more challenging as compared to more traditional designs.

The current study also has some limitations. One is the unbalanced group size caused by a higher patient recruitment rate during the intervention phase. This could be related to the stepped-wedge design and the GPs’ and PTs’ workshop participation, which likely increased the attention towards OA patients and access to PT-led OA education and exercise groups. We do not think this has influenced the generalisability of the sample, but because the patient participants were identified by their GPs and PTs, recruitment bias may potentially exist. Furthermore, self-reported QI pass rates, referrals, and body weight may be somewhat inaccurate due to recall bias and misconception, but the inaccuracy is likely to be similar across the groups. However, patients in the intervention group may have overestimated their physical activity levels more than those in the control group, potentially leading to bias.

Clinically effective and cost-effective treatments applied to large numbers of people with OA could result in substantial population health gains and reduced costs. We have demonstrated the uptake of recommended treatments in the SAMBA model in routine clinical practice. Clear estimates of the potential for clinical effectiveness and cost-effectiveness of the SAMBA model of care from the patients’ and societal perspectives are still needed.

### Implications for clinicians, policy makers, and future research

People with hip and knee OA represent a large, common patient group for GPs and PTs, and the provision of local, interdisciplinary workshops may facilitate multidisciplinary collaboration and ensure delivery of consistent patient information. When GPs and PTs are guided in the steps of structured, evidence-based care models, people with hip or knee OA may receive care that is more in line with current recommendations for OA care. Previous research has shown that people with knee OA have an almost 2-fold increased risk of sick leave and a 40%–50% increased risk of disability pension [47], and that the costs of hip and knee OA are substantial [48]. Hence, policy makers could consider facilitation of early secondary prevention strategies aiming to reduce the burden of the disease, which may potentially reduce the direct and indirect costs for the individual and the society. In addition, facilitation of treatment in groups (e.g., patient education and exercise sessions) represents an opportunity to provide access to care for more patients and to lower the costs compared to treating individual patients. A further improvement of the structured model may be included in future research, as well as exploring ways to implement the structured model for integrated OA care on a larger scale. The recent focus worldwide on healthcare overuse behaviours could be utilised to strengthen the work on preventing overuse of unnecessary treatments in OA care (e.g., opioids and imaging).

## Conclusions

This study demonstrated that a structured care model among GPs and PTs in primary healthcare improved the quality of care for patients with hip and knee OA. The model may be adapted to other chronic diseases treated in primary healthcare.

## Supporting information

S1 Text CONSORT checklist. (DOCX)

S2 Text TIDieR checklist. (PDF)

S3 Text Strategy to facilitate the use of the SAMBA model. (DOCX)

S4 Text OsteoArthritis Quality Indicator questionnaire version 2 (OA-QI v2). (DOCX)

## Abbreviations

BMI: body mass index
cluster-RCT: cluster-randomised controlled trial
GP: general practitioner
ICC: intraclass correlation coefficient
MRI: magnetic resonance imaging
OA: osteoarthritis
OA-QI v[number]: OsteoArthritis Quality Indicator questionnaire version [number]
OR: odds ratio
PT: physiotherapist
QI: quality indicator
SD: standard deviation
