# Correction: Implementing a structured model for osteoarthritis care in primary healthcare: A stepped-wedge cluster-randomised trial

**DOI:** 10.1371/journal.pmed.1002993

**Published:** 2019-12-19

**Authors:** Nina Østerås, Tuva Moseng, Leti van Bodegom-Vos, Krysia Dziedzic, Ibrahim Mdala, Bård Natvig, Jan Harald Røtterud, Unni-Berit Schjervheim, Thea Vliet Vlieland, Øyvor Andreassen, Jorun Nystuen Hansen, Kåre Birger Hagen

The third author’s name, Leti van Bodegom-Vos, is tagged incorrectly. As a result, the third author’s name is indexed incorrectly in the citation line and in PubMed. The correct initials are: van Bodegom-Vos L. The correct citation is: Østerås N, Moseng T, van Bodegom-Vos L, Dziedzic K, Mdala I, Natvig B, et al. (2019) Implementing a structured model for osteoarthritis care in primary healthcare: A stepped-wedge cluster-randomised trial. PLoS Med 16(10): e1002949. https://doi.org/10.1371/journal.pmed.1002949
