# Supplementary material for: Implementing a structured model for osteoarthritis care in primary healthcare: A stepped-wedge cluster-randomised trial
Source: PLoS Med. 2019 Oct 15;16(10):e1002949. doi: 10.1371/journal.pmed.1002949 (PMC6793845; doi:10.1371/journal.pmed.1002949)
Supplement: S1 Text — (DOCX) [file pmed.1002949.s001.docx]

**Modified CONSORT 2010 checklist of information to include when reporting a cluster randomised trial with extensions for cluster and stepped wedge design**

| Section/Topic | Item No | Standard Checklist item | Extension for cluster designs | Extension for stepped wedge | Page No * |
| --- | --- | --- | --- | --- | --- |
| Title and abstract | | | |  |  |
|  | 1a | Identification as a randomised trial in the title | Identification as a cluster randomised trial in the title | Identification as a stepped wedge cluster randomised trial | Title, Front page |
|  | 1b | Structured summary of trial design, methods, results, and conclusions (for specific guidance see CONSORT for abstracts)^[[1]](#endnote-1),^^[[2]](#endnote-2)^ | See table 2 |  | Abstract, Methods and findings + Conclusions |
| Introduction | | | |  |  |
| Background and objectives | 2a | Scientific background and explanation of rationale | Rationale for using a cluster design | Rationale for using stepped wedge design | Methods, Design, setting and participants, 1st para+ Study protocol  Discussion, Strengths and limitations |
|  | 2b | Specific objectives or hypotheses | Whether objectives pertain to the cluster level, the individual participant level or both |  | Introduction, last para + Study protocol |
| Methods | | | |  |  |
| Trial design | 3a | Description of trial design (such as parallel, factorial) including allocation ratio | Definition of cluster and description of how the design features apply to the clusters | Cohort (repeated measures on individuals), cross-sectional design (different  individuals), or mixture (open cohort)  Schematic representation of the trial design | Methods, Design, setting and participants, 1st para, Fig 1, Study protocol  Fig 1 |
|  | 3b | Important changes to methods after trial commencement (such as eligibility criteria), with reasons |  |  | n/a |
| Participants | 4a | Eligibility criteria for participants | Eligibility criteria for clusters |  | Methods, Design, setting and participants, 4th para |
|  | 4b | Settings and locations where the data were collected |  |  | Methods, Design, setting and participants, 1st para |
| Interventions | 5 | The interventions for each group with sufficient details to allow replication, including how and when they were actually administered | Whether interventions pertain to the cluster level, the individual participant level or both |  | Methods, Intervention + Fig 2 |
| Outcomes | 6a | Completely defined pre-specified primary and secondary outcome measures, including how and when they were assessed | Whether outcome measures pertain to the cluster level, the individual participant level or both |  | Methods, Data collection |
|  | 6b | Any changes to trial outcomes after the trial commenced, with reasons |  |  | n/a |
| Sample size | 7a | How sample size was determined | Method of calculation, number of clusters(s) (and whether equal or unequal cluster sizes are assumed), cluster size, a coefficient of intracluster correlation (ICC or *k*), and an indication of its uncertainty | Allowance for clustering,  Allowance for any repeated measures on individuals | Methods, Sample size calculation  Methods, Sample size calculation |
|  | 7b | When applicable, explanation of any interim analyses and stopping guidelines |  |  | n/a |
| Randomisation: | | | |  |  |
| Sequence generation | 8a | Method used to generate the random allocation sequence |  |  | Methods, Randomization and blinding |
|  | 8b | Type of randomisation; details of any restriction (such as blocking and block size) | Details of stratification or matching if used |  | Methods, Randomization and blinding |
| Allocation concealment mechanism | 9 | Mechanism used to implement the random allocation sequence (such as sequentially numbered containers), describing any steps taken to conceal the sequence until interventions were assigned | Specification that allocation was based on clusters rather than individuals and whether allocation concealment (if any) was at the cluster level, the individual participant level or both |  | Methods, Randomization and blinding |
| Implementation | 10 | Who generated the random allocation sequence, who enrolled participants, and who assigned participants to interventions | Replace by 10a, 10b and 10c |  |  |
|  | 10a |  | Who generated the random allocation sequence, who enrolled clusters, and who assigned clusters to interventions |  | Methods, Randomization and blinding |
|  | 10b |  | Mechanism by which individual participants were included in clusters for the purposes of the trial (such as complete enumeration, random sampling) |  | Methods, Randomization and blinding + Fig 1 |
|  | 10c |  | From whom consent was sought (representatives of the cluster, or individual cluster members, or both), and whether consent was sought before or after randomisation |  | Methods, Design, setting and participants, 2^nd^ para |
|  |  |  |  |  |  |
| Blinding | 11a | If done, who was blinded after assignment to interventions (for example, participants, care providers, those assessing outcomes) and how |  |  | Methods, Randomization and blinding |
|  | 11b | If relevant, description of the similarity of interventions |  |  | n/a |
| Statistical methods | 12a | Statistical methods used to compare groups for primary and secondary outcomes | How clustering was taken into account | Allowance for clustering (that is, random effect model). Allowance for repeated measures on individuals, if appropriate. | Methods, Statistical analyses |
|  | 12b | Methods for additional analyses, such as subgroup analyses and adjusted analyses |  |  | n/a |
| Results | | | |  |  |
| Participant flow (a diagram is strongly recommended) | 13a | For each group, the numbers of participants who were randomly assigned, received intended treatment, and were analysed for the primary outcome | For each group, the numbers of clusters that were randomly assigned, received intended treatment, and were analysed for the primary outcome |  | Results, 1^st^ para + Fig 1 + Fig 3 |
|  | 13b | For each group, losses and exclusions after randomisation, together with reasons | For each group, losses and exclusions for both clusters and individual cluster members |  | Results, 1^st^ para + Fig 3 |
| Recruitment | 14a | Dates defining the periods of recruitment and follow-up |  |  | Methods, Design, setting and participants, 1st para+ Fig 1 |
|  | 14b | Why the trial ended or was stopped |  |  | n/a |
| Baseline data | 15 | A table showing baseline demographic and clinical characteristics for each group | Baseline characteristics for the individual and cluster levels as applicable for each group | Characteristics of sample reported by exposed and unexposed observation  periods, or by randomisation group | Table 1 |
| Numbers analysed | 16 | For each group, number of participants (denominator) included in each analysis and whether the analysis was by original assigned groups | For each group, number of clusters included in each analysis |  | Results, 1^st^ para + Fig 3 |
| Outcomes and estimation | 17a | For each primary and secondary outcome, results for each group, and the estimated effect size and its precision (such as 95% confidence interval) | Results at the individual or cluster level as applicable and a coefficient of intracluster correlation (ICC or k) for each primary outcome | Adjusted (for time) treatment effect and 95% CI should be interpreted as  unbiased estimate of effect size  Schematic representation of actual study design  Intention to treat analysis should follow the randomised design and might be  different to that which actually transpired | Results, Primary outcome + Results, Secondary outcomes +Table 2-3  Fig 1  Results, three first para |
|  | 17b | For binary outcomes, presentation of both absolute and relative effect sizes is recommended |  |  |  |
| Ancillary analyses | 18 | Results of any other analyses performed, including subgroup analyses and adjusted analyses, distinguishing pre-specified from exploratory |  |  |  |
| Harms | 19 | All important harms or unintended effects in each group (for specific guidance see CONSORT for harms^[[3]](#endnote-3)^) |  |  | Results, 3^rd^ para |
| Discussion | | | |  |  |
| Limitations | 20 | Trial limitations, addressing sources of potential bias, imprecision, and, if relevant, multiplicity of analyses |  |  | Discussion, Strengths and limitations |
| Generalisability | 21 | Generalisability (external validity, applicability) of the trial findings | Generalisability to clusters and/or individual participants (as relevant) |  | Discussion, Strengths and limitations |
| Interpretation | 22 | Interpretation consistent with results, balancing benefits and harms, and considering other relevant evidence |  |  | Discussion, Primary outcome + Discussion, Secondary outcomes |
| Other information | | |  |  |  |
| Registration | 23 | Registration number and name of trial registry |  |  | Methods, Design, setting and participants, 1st para |
| Protocol | 24 | Where the full trial protocol can be accessed, if available |  |  | Methods, Design, setting and participants, 1st para + Reference #26 + Supporting information |
| Funding | 25 | Sources of funding and other support (such as supply of drugs), role of funders |  |  | Funding |

** Note: page numbers optional depending on journal requirements*

1. [↑](#endnote-ref-1)
2. [↑](#endnote-ref-2)
3. [↑](#endnote-ref-3)
