# Supplementary material for: Implementing a structured model for osteoarthritis care in primary healthcare: A stepped-wedge cluster-randomised trial
Source: PLoS Med. 2019 Oct 15;16(10):e1002949. doi: 10.1371/journal.pmed.1002949 (PMC6793845; doi:10.1371/journal.pmed.1002949)
Supplement: S3 Text — (DOCX) [file pmed.1002949.s003.docx]

**Appendix S3. Strategy to facilitate the use of the SAMBA model.**

| **Target group** | **Barrier** | **Activity** | **Description** |
| --- | --- | --- | --- |
| General practitioners | Awareness, knowledge, attitude, motivation to change, and behavioural routines | Workshop (provision of information) | The GPs received oral and written information on recommended OA care, the PT treatment programme, imaging modalities in OA, and information about the appropriate time to refer to an orthopaedic surgeon. The workshop was embedded in existing GP meetings, was interactive, and allowed time for discussions. SAMBA was presented as a useful ‘tool’. The multidisciplinary workshops ensured that the GPs met their neighbouring PTs, learned more about each others’ roles in OA care and enabled a multidisciplinary discussion regarding OA care. |
|  | Awareness, knowledge | Education material | The GPs received a summary of international guidelines for OA care. |
|  | Awareness, knowledge, attitude, motivation to change, and behavioural routines | Education outreach visits | All general practice clinics were visited twice during the intervention period. Each clinic received a reminder call quarterly by the project coordinator. |
|  | Awareness | Reminder material | Posters, pens, post-it note pads, and mouse mats were distributed during the workshop and during educational outreach visits. |
|  | Motivation to change | Opinion leaders/endorsement | Local opinion leaders were identified and asked to promote the intervention among their colleagues. The GPs’ association was asked to endorse the SAMBA model. |
|  | Awareness, motivation to change | Feedback, audit | Study newsletters were distributed 3 times a year. Feedback on recruitment rate was included. |
|  | Accessibility, attitude, behavioural change | Direct access to FLS and PT in private practice | The PT working at FLS or in private practice was asked to prioritize the SAMBA patients by ensuring a quick initial assessment and enrolment in the OA programme. |
| Physiotherapists at FLS and in private practice | Awareness, knowledge, attitude, motivation to change and behavioural routines | Workshop (provision of information) | The PTs were educated in delivering OA care in accordance with clinical guidelines with a standardized patient education material and exercise programme recommendations for patients with OA symptoms primarily from the hip or knee + how to adapt the standard modes of delivery to the needs of the individual OA patient. The multidisciplinary workshops ensured that the PTs met their neighbouring GTs, learned more about each others’ roles in OA care and enabled a multidisciplinary discussion regarding OA care. |
|  | Awareness, knowledge | Education material | The PTs received a summary of international guidelines for non-pharmacological OA care. |
|  | Awareness, knowledge, attitude, motivation to change, and behavioural routines | Education outreach visits | All FLSs and private PT practices were visited twice during the intervention period. Each clinic received a follow-up call quarterly by the project coordinator. |
|  | Awareness | Reminder material | Posters, pens, and post-it note pads were distributed during the workshop and during educational outreach visits. |
|  | Motivation to change | Feedback, audit | Study newsletters were distributed 3 times a year. |
|  | Awareness, motivation to change | Endorsement, continuing educational points | The Norwegian Physiotherapist Association was asked to endorse the ActiveA programme and to provide accreditation of the workshop for continuing educational points. |
