# Supplementary figures and images for: Implementing a structured model for osteoarthritis care in primary healthcare: A stepped-wedge cluster-randomised trial
Source: PLoS Med. 2019 Oct 15;16(10):e1002949. doi: 10.1371/journal.pmed.1002949 (PMC6793845; doi:10.1371/journal.pmed.1002949)

**S1. OsteoArthritis Quality Indicator questionnaire v2 (OA-QIv2)**

**
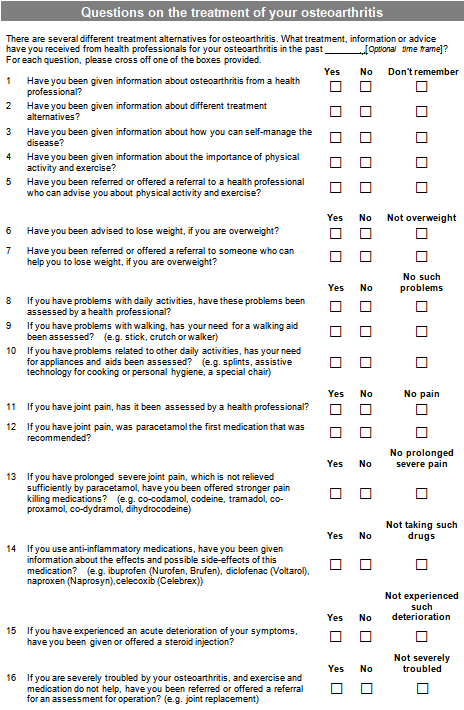
**

Supplement: S4 Text — (DOCX) [file pmed.1002949.s004.docx]
